# B-type natriuretic peptide is neither itch-specific nor functions upstream of the GRP-GRPR signaling pathway

**DOI:** 10.1186/1744-8069-10-4

**Published:** 2014-01-18

**Authors:** Xian-Yu Liu, Li Wan, Fu-Quan Huo, Devin M Barry, Hui Li, Zhong-Qiu Zhao, Zhou-Feng Chen

**Affiliations:** 1 Center for the Study of Itch, Washington University School of Medicine Pain Center, St. Louis, MO 63110, USA; 2 Departments of Anesthesiology, Washington University School of Medicine Pain Center, St. Louis, MO 63110, USA; 3 Departments of Psychiatry, Washington University School of Medicine Pain Center, St. Louis, MO 63110, USA; 4 Departments of Developmental Biology, Washington University School of Medicine Pain Center, St. Louis, MO 63110, USA

**Keywords:** BNP, NPRA, GRP, GRPR, Itch, Pain, Spinal cord, DRG

## Abstract

**Background:**

A recent study by Mishra and Hoon identified B-type natriuretic peptide (BNP) as an important peptide for itch transmission and proposed that BNP activates spinal natriuretic peptide receptor-A (NPRA) expressing neurons, which release gastrin releasing peptide (GRP) to activate GRP receptor (GRPR) expressing neurons to relay itch information from the periphery to the brain (*Science* 340:968–971, 2013). A central premise for the validity of this novel pathway is the absence of GRP in the dorsal root ganglion (DRG) neurons. To this end, they showed that *Grp* mRNA in DRG neurons is either absent or barely detectable and claimed that BNP but not GRP is a major neurotransmitter for itch in pruriceptors. They showed that NPRA immunostaining is perfectly co-localized with *Grp*-eGFP in the spinal cord, and a few acute pain behaviors in *Nppb*^*-/-*^ mice were tested. They claimed that BNP is an itch-selective peptide that acts as the first station of a dedicated neuronal pathway comprising a GRP-GRPR cascade for itch. However, our studies, along with the others, do not support their claims.

**Findings:**

We were unable to reproduce the immunostaining of BNP and NPRA as shown by Mishra and Hoon. By contrast, we were able to detect *Grp* mRNA in DRGs using *in situ* hybridization and real time RT-PCR. We show that the expression pattern of *Grp* mRNA is comparable to that of GRP protein in DRGs. Pharmacological and genetic blockade of GRP-GRPR signaling does not significantly affect intrathecal BNP-induced scratching behavior. We show that BNP inhibits inflammatory pain and morphine analgesia.

**Conclusions:**

Accumulating evidence demonstrates that GRP is a key neurotransmitter in pruriceptors for mediating histamine-independent itch. BNP-NPRA signaling is involved in both itch and pain and does not function upstream of the GRP-GRPR dedicated neuronal pathway. The site of BNP action in itch and pain and its relationship with GRP remain to be clarified.

## Background

Identification of novel itch mediators in sensory neurons and in the spinal cord and elucidation of the molecular and cellular circuitry have become a new and exciting frontier for the past several years. As many itch mediators (neuropeptides, receptors and channels etc.) have been implicated in itch transmission, conflicting results and confusion emerge. Gastrin releasing peptide (GRP) is a neuropeptide expressed in primary afferents and is necessary for relaying non-histaminergic itch from the skin to the spinal cord [1-4]. For several decades, the availability of the antibody against the highly conserved nine amino acids in the C terminus of GRP/bombesin across species has enabled the demonstration of expression of GRP in primary afferents of rat, mouse, cat, monkey and human. Using the anti-GRP or bombesin antibody, the percentage of GRP^+^ cells has been consistently estimated to be around 5–8% in dorsal root ganglion (DRG) neurons and 12% in trigeminal ganglia (TG) neurons of rodents [2,5-14]. Several groups have used dorsal root rhizotomy to examine the origin of GRP^+^ primary afferents. For the past 30 years, all the studies have consistently found that the majority of GRP^+^ fibers are derived from primary afferents [2,4,7-9,15], with only one exception [16]. The earlier findings on the origin of GRP^+^ fibers have been reviewed previously [1,17]. In addition, neonatal capsaicin treatment dramatically depleted GRP^+^ fibers in the spinal cord, which supported the notion that the source of GRP is from primary afferents [18]. It is important to note that the specificity of the anti-GRP antibody has been repeatedly confirmed using *Grp*^-/-^ mice [4,19] and by an antigen absorption approach [16], the latter of which was first performed more than 30 years ago [8]. Importantly, several studies found that GRP expression was markedly enhanced in primary afferents in mice, monkeys and patients with various chronic itch conditions [4,11-13], matching an up-regulation of GRPR in the spinal cord [4,12]. Those studies support a physiological role of GRP in primary afferents. On the other hand, the specific expression of *Grp* mRNA in lamina I of the spinal cord and a failure to detect *Grp* mRNA in DRG neurons using *in situ* hybridization (ISH) by some investigators have reignited interests in spinal *Grp* expression [16]. To date, little evidence exists to support that *Grp* mRNA in lamina I of the spinal cord is in fact translated into GRP protein because none of the GRP immunohistochemistry studies after dorsal root rhizotomy have revealed the neuronal cell body staining pattern that resembles that of *Grp* mRNA in the spinal cord. It was speculated that some of the residual GRP^+^ fiber staining in the spinal cord after rhizotomy could be of descending origins [8,9]. Moreover, an up-regulation of GRP protein intrinsic to the dorsal horn in the setting of chronic itch was not detected [4], further arguing against the physiological role of *Grp* mRNA in the spinal cord. Additional evidence supporting the idea of activation of postsynaptic GRPR by presynaptic GRP release has also emerged. Potential synaptic contacts between MrgprA3^+^ primary afferents and GRPR^+^ neurons were demonstrated [20]. Electron microscopic analysis revealed that GRP^+^ terminals contained large dense-core vesicles that formed synaptic contacts with a few dendrites of dorsal horn neurons [15]. Despite a large body of literature that demonstrated the presence of GRP protein in DRGs and primary afferents, a recent study by Mishra and Hoon attempted to show a lack of *Grp* mRNA in sensory neurons and proposed that B-type natriuretic peptide (BNP), rather than GRP, is a major neuropeptide in primary afferents for itch transmission [21]. Further, they proposed that BNP activates natriuretic peptide receptor-A (NPRA) expressing neurons in the spinal cord, which in turn release GRP to activate GRPR^+^ neurons. In this report, we present the data that do not support their conclusions.

### NPRA and BNP expression in DRGs and spinal cord

**Figure 1:**
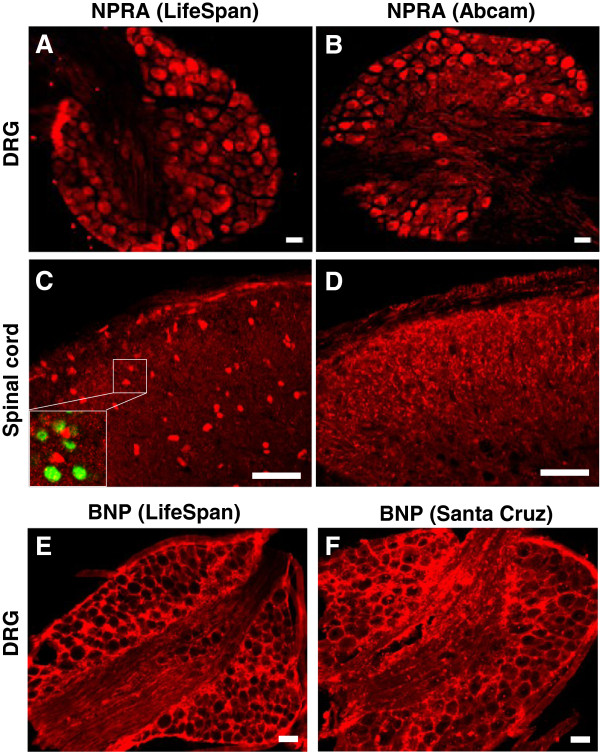
**NPRA and BNP immunostaining in DRG neurons and spinal cord. A-D)** NPRA immunostaining in mouse DRG (upper row) and dorsal spinal cord (lower row) was performed with antibodies from LifeSpan BioSciences **(A, C)** and Abcam **(B, D)**. The inset in **(C)** shows non-specific signals that do not overlap with NeuN staining (green). **E-F)** BNP immunostaining in DRG using antibodies from LifeSpan Biosciences **(E)** and Santa Cruz **(F)**. Scale bars: 50 μm.

BNP, which is encoded by the *Nppb* gene, and its receptor NPRA that is encoded by *Npr1* have been studied for decades [22] and recently implicated in itch transmission [21]. Using *Grp*-eGFP BAC-transgenic mice, Mishra and Hoon reported a perfect co-localization of NPRA protein and eGFP expression in lamina I of the spinal cord using a LifeSpan antibody for NPRA. However, this striking image is inconsistent with two additional findings: 1) they showed that *Npr1*^+^ cells are scattered throughout the dorsal horn, whereas several studies including our own have demonstrated that *Grp* mRNA is largely restricted to lamina I [23-25]; 2) with an Abcam antibody, no laminae I-II-restricted NPRA^+^ immunostaining was detected after dorsal root rhizotomy to remove intense NPRA^+^ fibers in the dorsal horn of rats [26]. Indeed, immunostaining using the two different antibodies shows comparable widespread NPRA^+^ expression pattern in DRG neurons (Figure 1A, 1B), in accordance with studies in mice and in rats [26,27]. However, in the spinal cord no lamina-specific NPRA^+^ staining was observed using the LifeSpan antibody (Figure 1C) as reported by Mishra and Hoon, while an Abcam antibody reveals intense NPRA^+^ primary fibers (Figure 1D), reminiscent of the finding in rat [26]. Our comparative studies suggest that the Abcam NPRA antibody may better recapitulate endogenous expression of NPRA in sensory neurons.

Mishra and Hoon showed BNP immunostaining in mouse DRG neurons [21]. Using the same antibody (LifeSpan) and another BNP antibody from Santa Cruz, however, we found satellite glial like pattern in DRGs (Figure 1E, 1F). Using a different BNP antibody (Phoenix Pharmaceuticals), another group recently found that BNP immunostaining was similarly present in non-neuronal cells and rarely detected in neuron-like cells in mouse TGs [27], suggesting a possible involvement of peripheral BNP in neuron-glia crosstalk. Although these results are not consistent with ISH studies [21] in DRG neurons nor with the data in rats [26], three BNP antibodies supplied by different companies all showed non-neuronal cell-like staining in DRGs or TGs of mice. In light of these discrepancies, it will be helpful to verify the specificity of BNP antibodies in *Nppb*^-/-^ mice and to define whether BNP protein is expressed in primary afferents.

### *Grp* mRNA expression in DRG neurons

**Figure 2:**
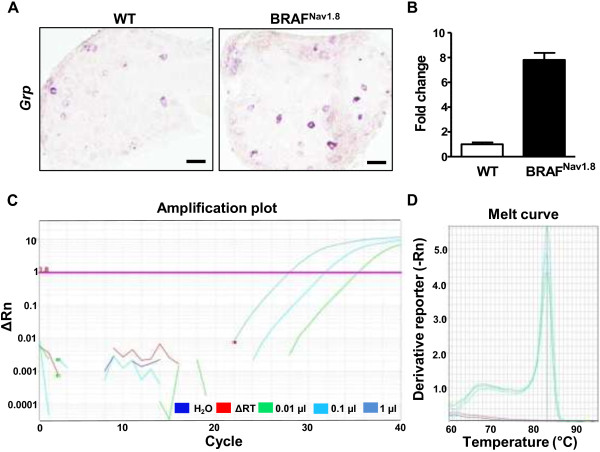
**Expression of *Grp *mRNA in DRGs. A)***Grp* mRNA was detected in WT and BRAF^Nav1.8^ DRGs using ISH. Scale bars: 100 μm. **B)** Real-time RT-PCR showed that *Grp* mRNA level was dramatically enhanced in BRAF^Nav1.8^ DRG. n = 4. *p < 0.0001 versus WT, unpaired t test. **C)** Validation of *Grp* primers using real-time RT-PCR and a series of amounts of DRG cDNA (0.01, 0.1 and 1 μl). The reactions were specific because there were no detectable signals when DRG cDNA was substituted with H_2_O (blue) or ∆RT control (red) in which reverse transcriptase was omitted. **D)** Melt curve to show that *Grp* PCR product has a single narrow peak at 83°C.

A central premise for the validity of the BNP-NPRA-GRP-GRPR cascade proposed by Mishra and Hoon is a lack of GRP in primary afferents. Although a specific GRP antibody has eliminated the necessity for examining *Grp* mRNA using ISH technique, single cell RT-PCR permits a more precise assessment of co-expression of *Grp* mRNA with other markers such as *Trpv1* mRNA in a subset of DRG neurons [28,29]. Instead of using anti-GRP antibody or single cell RT-PCR, Mishra and Hoon presented three pieces of evidence to dispute the presence of *Grp* mRNA in DRGs [21]. First, they could not detect *Grp* mRNA in DRGs using ISH. Unfortunately, *Grp* mRNA detection in DRGs by ISH has remained a challenge for some investigators [16,21]. We have recently shown that BRAF^Nav1.8^ mice represent a unique animal model for investigating the mechanisms of chronic itch [4]. In BRAF^Nav1.8^ mice, the expression of a cohort of itch-related mediators including GRP protein was markedly increased in DRGs, which account for the development of spontaneous itch in these mice [4]. Using ISH, we detected a few *Grp*^*+*^ cells in wild-type (WT) DRGs, consistent with GRP immunostaining pattern (Figure 2A). In contrast, many more *Grp*^+^ cells were detected in DRGs of BRAF^Nav1.8^ mice, demonstrating enhanced *Grp* mRNA expression in the setting of chronic itch (Figure 2A), consistent with enhanced GRP protein expression as detected using anti-GRP antibody [4]. These conflicting results indicate that the sensitivity of ISH rests on multiple factors (e.g. tissues, age of animals, perfusion method, the abundance of transcripts, etc.). Concerning gene expression in DRGs and spinal cord, it is not uncommon that mRNA or protein for some genes can be easily detected in one tissue but not the other. Therefore, ISH procedure or immunostaining procedure has to be optimized and tailored to specific cRNA probes/antibodies if the routine procedure fails to work.

Second, Mishra and Hoon tried to minimize the presence of *Grp* mRNA by stating that they were unable to detect more than “trace quantities” of *Grp* expression in DRGs using a “sensitive quantitative” PCR assay [21]. However, “trace quantities” of *Grp* could be a sufficient amount of mRNA to generate physiologically relevant level of GRP that is necessary for GRPR activation in the spinal cord, particularly given that GRP can bind and activate GRPR at nanomolar concentrations [30]. Using real-time RT-PCR and validated *Grp* primers, we detected a dramatic up-regulation of *Grp* mRNA in BRAF^Nav1.8^ DRGs (Figure 2B), in line with the ISH results (Figure 2A). *Grp* cDNA was amplified from as low as 0.01 μl of WT DRG cDNA while no amplification was seen when using up to 1 μl of control samples (∆RT) in which reverse transcriptase was omitted (Figure 2C). The single narrow peak at 83°C in melt curve further validated the high quality of the reactions (Figure 2D).

Finally, Mishra and Hoon used *Grp*-eGFP transgenic mice generated by GENSAT as evidence to argue for an absence of *Grp* mRNA expression in DRGs. It has been well known that transgenic eGFP mouse lines generated by BAC engineering-based technique may not necessarily recapitulate endogenous expression as over-expression or an absence of eGFP expression are two frequent problems encountered, which can be attributable to the integration site and copy number of the transgene in the mouse genome and the size of the genomic DNAs used to construct the vector. Although BAC-based transgenic lines serve as a great tool for researchers to follow endogenous expression of many genes, it is important to validate the line using either functional or molecular approaches. A common practice is to screen the transgenic mice derived from pronuclear injection of a BAC vector and to identify the founder that may best mimic the endogenous gene expression, since there are great variations of transgene expression among the transgenic mice. One can find more information at GENSAT website: http://www.gensat.org/index.html about the discrepancies between eGFP expression patterns generated by GENSAT and those in literature. It was also not rare to see discrepancies in the eGFP expression patterns for the same gene between different mouse lines when different BAC vectors were used. For example, two independent lines of *Grp-cre* mice (KH-107 and KH-288) generated by GENSAT exhibit distinct and overlapping expression patterns in some areas of the brain. Therefore, the expression pattern of a transgenic line cannot be considered as valid evidence to argue for an absence of the expression of a gene.

### BNP functions independent of GRP and GRPR

**Figure 3:**
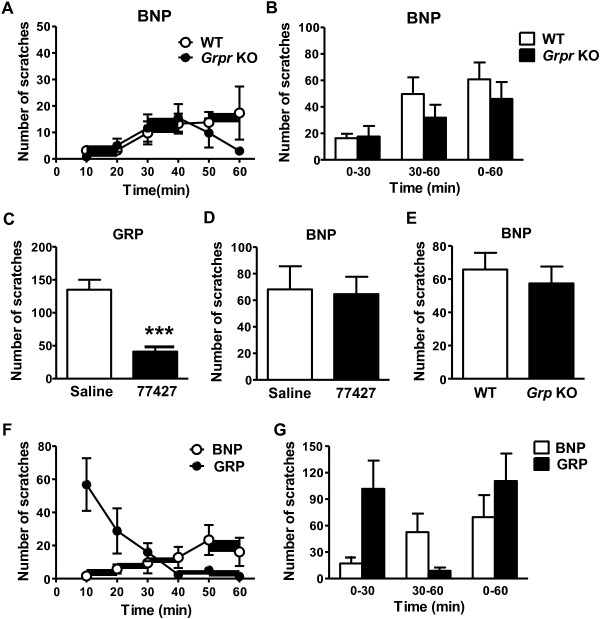
**BNP functions independently of GRPR. A, B)** BNP (1 nmol, i.t.) induced comparable scratching behavior in WT and *Grpr* KO mice as shown in 10 min time-course **(A)** and total **(B)**. n = 6. **C, D)** Co-injection of GRP inhibitor 77427 (100 nmol, i.t.) significantly blocked 1 nmol GRP-induced scratching behavior **(C)** while it had no effect on scratching behavior induced by 1 nmol BNP **(D)**. n = 5. ***p < 0.001 versus saline, unpaired t test. **E)** BNP-induced scratching behavior did not differ between WT and *Grp* KO mice. n = 7–10. **F, G)** Comparison of scratching behaviors induced by i.t. injection of BNP and GRP as shown in 10 min time-course **(F)** and total **(G)**. n = 7.

If BNP-NPRA signaling depends on the activation of GRPR by GRP, intrathecal (i.t.) BNP-elicited scratching behavior would be lost or attenuated in *Grpr* knock-out (KO) or *Grp* KO mice. However, we did not observe significant difference in BNP-elicited scratching responses between *Grpr* KO mice and their WT littermates (Figure 3A, 3B). Next we examined whether i.t. BNP-induced scratching behavior could be attenuated by 77427 (Chembridge), a GRP blocker whose specificity was recently confirmed [4]. 77427 effectively reduced i.t. GRP-induced scratching behavior (Figure 3C). In contrast, i.t. BNP-induced scratching behavior was not inhibited by co-injection of 77427 (Figure 3D). Similarly, *Grp* KO mice and their WT littermates showed comparable scratching behavior evoked by i.t. BNP (Figure 3E). These genetic and pharmacological blockade studies demonstrate that GRP-GRPR signaling does not act downstream of BNP in relaying itch information. Notably, the time-course of scratching responses evoked by i.t. BNP and GRP differs significantly. In WT mice, i.t. BNP (1 nmol) induced mild bilateral scratching behavior in the first 30 min and the scratching behavior was gradually increased in the second 30 min during the one-hour observation (Figure 3F, 3G). In contrast, i.t. GRP (1 nmol) induced robust scratching responses for about 10 min after injection and the number of scratching bouts decreased gradually within 40 min. Nonetheless, these interesting and apparent differences in pharmacokinetics of the two peptides further argue against the possibility that BNP and GRP function in the same pathway.

### Intrathecal BNP inhibits inflammatory pain and morphine analgesia

Mishra and Hoon tested a few acute pain behaviors in *Nppb*^*-/-*^ mice and proposed that BNP is an “itch-selective” neuropeptide that acts as the first station in primary afferents to activate the GRP-GRPR dedicated neuronal pathway for itch via spinal NPRA [21]. However, BNP and NPRA in DRG neurons have been suggested to be a novel auto-feedback nociceptive pathway to modulate inflammatory pain [26] and BNP is a negative regulator of neuronal excitability in response to nociceptive stimuli [27]. Moreover, i.t. BNP attenuated morphine analgesia in mice [31].

**Figure 4:**
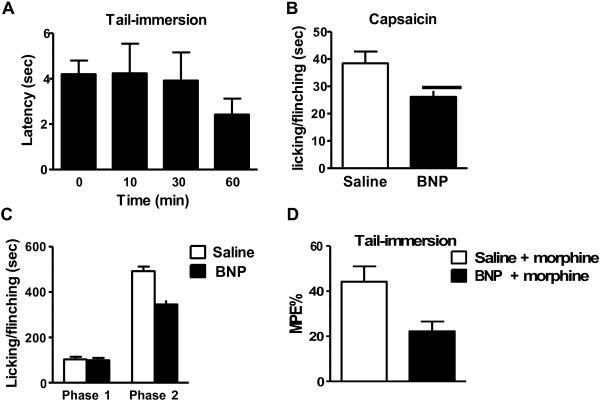
**BNP inhibits inflammatory pain. A)** BNP (0.04 nmol, i.t.) did not alter the baseline of thermal sensation as tested in a 50°C tail-immersion assay. n = 9, p > 0.05, one-way ANOVA. **B)** BNP significantly reduced the licking and flinching time induced by capsaicin (2 μg, i.pl.). n = 10. *p < 0.05 versus saline, unpaired t test. **C)** BNP (0.1 nmol, i.t.) did not change the first phase (0–10 min), but significantly reduced the second phase (10–60 min) of formalin pain. n = 6–7. *p < 0.05 versus saline, unpaired t test. **D)** Pre-injection of BNP (0.04 nmol, i.t.) 10 min before morphine injection (5 mg/kg, i.p.) significantly blocked morphine analgesic effect as tested by tail-immersion assay 60 min after morphine injection. n = 6. *p < 0.05 versus pre-saline + morphine, unpaired t test.

We next examined the role of BNP in nociceptive processing. Although BNP (0.04 nmol) did not alter acute thermal pain as tested in 50°C tail-immersion assay (Figure 4A), it significantly reduced the licking and flinching time induced by intraplantar (i.pl.) injection of capsaicin (Figure 4B). I.t. BNP also markedly attenuated formalin-evoked nocifensive behavior during phase II response (Figure 4C) and reduced morphine analgesia (Figure 4D). These results confirm that BNP plays an important role in nociceptive processing in mice. In contrast, neither GRP nor GRPR is involved in morphine analgesia [32] and both loss- and gain-of-function studies consistently demonstrated that GRP-GRPR signaling is not involved in nociceptive processing [2-4,32]. Therefore, the BNP-NPRA and GRP-GRPR pathways are unlikely to be in the same dedicated neuronal pathway for itch.

## Conclusions

The present study questions the experimental approaches that Mishra and Hoon used and the data they presented in their studies. Specifically, we question the BNP immunostaining pattern in DRGs as we could not recapitulate their results in DRG neurons using the same antibody. For NPRA staining, we detected numerous NPRA^+^ neurons in DRGs and NPRA^+^ fibers in the spinal cord and were unable to detect lamina I-restricted NPRA^+^ neuronal staining pattern that resembles that of *Grp* mRNA expression in the spinal cord. We show that BNP-NPRA signaling is involved in both itch and pain, which contrasts with itch-specific GRP-GRPR signaling. Given that BNP could activate peripheral NPRA to reduce the excitability of sensory neurons and the conflicting data on BNP expression in sensory neurons [26,27], we suggest that the sites of action (glia vs neurons; DRGs vs spinal cord) and the underlying mechanisms (inhibitory vs excitatory) for BNP and NPRA in itch and pain remain to be clarified. We demonstrate that *Grp* mRNA is detectable by ISH and by real-time RT-PCR in DRGs. More important, we demonstrate that BNP-NPRA signaling does not function as the first station upstream of the GRP-GRPR pathway using both pharmacological and genetic approaches, the latter of which is considered to be superior to the former. Taken together, abundant evidence supports the notion that GRP is indeed a primary afferent transmitter for itch and argues against the linear BNP-NPRA-GRP-GRPR cascade. Nevertheless, identification of BNP in itch transmission provides an important and new avenue for further investigating itch signaling from the pruriceptors to the spinal cord. The action of BNP-NPRA in itch and pain and their relationship with GRP-GRPR signaling require more extensive investigation.

## Materials and methods

### Animals

Male mice between 7 and 12 weeks old were used for the experiments. C57BL/6 J mice were purchased from the Jackson Laboratory. *Grp KO mice*, *Grpr* KO mice and BRAF^Nav1.8^ mice were used as described [2,4]. All the mice were housed under a 12 h light/dark cycle with food and water provided *ad libitum*. All the animal experiments were performed in accordance with the guidelines of the National Institutes of Health and the International Association for the Study of Pain and were approved by the Animal Studies Committee at Washington University School of Medicine.

### Immunohistochemistry and ISH

Immunohistochemical staining was performed as described [33]. Briefly, mice were anesthetized with an overdose of ketamine/xylazine cocktail and fixed by intracardiac perfusion of cold 0.01 M PBS (pH 7.4) and 4% paraformaldehyde. Tissues were immediately removed, post-fixed in the same fixative overnight at 4°C, and cryoprotected in 30% sucrose PBS solution. Tissues were frozen and sectioned at 20–25 μm thickness on a cryostat. Free-floating sections were blocked in a solution containing 2% donkey serum and 0.3% Triton X-100 in PBS for 1 h at room temperature. The sections were incubated with primary antibodies overnight at 4°C followed by secondary antibodies. The following primary antibodies were used at the specified dilutions: rabbit anti-NPRA (1:200, LS-C109432, LifeSpan Bioscience), rabbit anti-NPRA (1:300, ab70848, Abcam), mouse anti-BNP (1:100, LS-C82084, LifeSpan Bioscience), mouse anti-NeuN (1:10,000, MAB377, Millipore), and goat anti-BNP (V-17) (1:100, sc-67455, Santa Cruz). The secondary antibodies were purchased from Jackson ImmunoResearch Laboratories, Inc. including Cy3- or FITC-conjugated donkey anti-rabbit or anti-mouse IgG (1:1,000). For the ISH study, a digoxigenin-labeled cRNA probe was used as described earlier [34]. Images were taken using a Nikon Eclipse Ti-U microscope and confocal microscope (Leica TCS SPE).

### Real-time RT-PCR

Real-time RT-PCR was performed as previously described [32]. Briefly, DRGs were dissected out from 9 weeks old male mice (n = 4 per genotype). Tissues were temporarily stored on ice in 1 ml of RNA stabilizer (RNAlater, QIAGEN). Total RNA was isolated and genomic DNA was removed in accordance with manufacturer’s instructions (RNeasy plus mini kit; QIAGEN). RNA was quantified using Eppendorf BioPhotometer and stored at -80°C. Single-stranded cDNA was synthesized from 1 μg of total RNA using High Capacity cDNA Reverse Transcription Kit (Life Technologies) and stored at -20°C until the analysis. Reverse transcriptase was omitted for negative control (∆RT). Gene expression of *Grp* was determined by real-time PCR (StepOnePlus; Applied Biosystems). Specific primers spanning intron were designed with the NCBI Primer-BLAST. The fidelity and specificity of the primers were validated by real-time PCR using serial volume (1 μl, 0.1 μl and 0.01 μl) of WT DRG cDNA and PCR efficiency (Ef) was calculated. The primers used are:

*Grp* (NM_175012.2, Ef = 0.8632, R^2^ = 0.9990): 5′-TGGGCTGTGGGACACTTAAT-3′ (exon1); 5′-GCTTCTAGGAGGTCCAGCAAA-3′ (exon2); amplicon size: 146 nt; intron size: 6,158 bp.

*Actb* (NM_007393.3, Ef = 0.9987, R^2^ = 1): 5′-TGTTACCAACTGGGACGACA-3′ (exon3); 5′-GGGGTGTTGAAGGTCTCAAA-3 (exon 4)′; amplicon size: 166 nt. intron size: 454 bp.

Real-time PCR was carried out with FastStart Universal SYBR Green Master (Roche Applied Science). All samples (0.1 μl) were assayed in duplicates. Thermal cycling was initiated with denaturation at 95°C for 10 min. After this initial step, 40 cycles of PCR (heating at 95°C for 10 sec and 60°C for 30 sec) were performed. Data were analyzed using Comparative CT Method (StepOne Software v2.2.2.) and the expression of *Grp* was normalized to the expression of *Actb* after adjustment with Ef.

### Behavioral tests

Behavioral tests were videotaped (HDR-CX190, Sony) from a side angle. The videos were played back on a computer and the quantification of mice behaviors were done by persons who were blinded to the treatments and genotypes.

#### Scratching behavior

Itch behaviors were performed as previously described [2,3]. Briefly, prior to experiments, mice were given 30 min to acclimate to the plastic arenas (10 × 10.5 × 15 cm). Mice were then briefly removed from the chamber for drug injections.

#### Thermal sensitivity

Thermal sensitivity was determined using tail immersion assay. Mice tails were dipped beneath the warm water (50°C) in a temperature-controlled water bath (IITC Inc.). The latency to withdrawal was measured with a 12-sec cutoff. For morphine analgesia study, BNP (Phoenix Pharmaceuticals) was intrathecally injected 10 min before morphine injection (5 mg/kg, i.p.) and tail immersion (50°C) results were expressed as percentage of maximum possible effect [%MPE = (post drug latency - pre drug latency) × 100/ (cutoff time – pre drug latency)].

#### Acute nociceptive behavior

Algogens were intraplantarly injected into right hindpaws. The duration of licking and flinching of the injected paw was recorded for 60 min after injection of formalin (2%, 20 μl) and for 5 min after injection of capsaicin (2 μg, 20 μl).

### Data analysis

All values are expressed as the means ± standard error of the mean (S.E.M.). Statistical analysis was performed using Prism v5.03 (GraphPad Software). p < 0.05 was considered statistically significant.

## Abbreviations

BNP: B-type natriuretic peptide; NPRA: Natriuretic peptide receptor-A; GRP: Gastrin releasing peptide; GRPR: Gastrin releasing peptide receptor; ISH: *In situ* hybridization; DRG: Dorsal root ganglion; TG: Trigeminal ganglions; i.t.: intrathecal.

## Competing interests

The authors declare that they have no competing interests.

## Authors’ contributions

XYL performed real-time RT-PCR, LW and ZQZ performed behavioral experiments, FQH, DB, and HL performed immunostaining and ISH, ZFC supervised the project and ZFC and XYL wrote the manuscript. All authors read and approved the final manuscript.
